# 1-(Prop-2-yn­yl)-1*H*-benzimidazol-2(3*H*)-one

**DOI:** 10.1107/S160053681205088X

**Published:** 2012-12-22

**Authors:** Younès Ouzidan, Youssef Kandri Rodi, Fouad Ouazzani Chahdi, El Mokhtar Essassi, Mohamed Saadi, Lahcen El Ammari

**Affiliations:** a Laboratoire de Chimie Organique Appliquée, Université Sidi Mohamed Ben Abdallah, Faculté des Sciences et Techniques, Route d’immouzzer, BP 2202 Fès, Morocco; b Laboratoire de Chimie Organique Hétérocyclique URAC21, Faculté des Sciences, Université Mohammed V-Agdal, Avenue Ibn Battouta, BP 1014, Rabat, Morocco; c Institute of Nanomaterials and Nanotechnology, MASCIR, Rabat, Morocco; d Laboratoire de Chimie du Solide Appliquée, Faculté des Sciences, Université Mohammed V-Agdal, Avenue Ibn Battouta, BP 1014, Rabat, Morocco

## Abstract

The benzimidazolone part of the title mol­ecule, C_10_H_8_N_2_O, is almost planar [r.m.s. deviation = 0.014 (1) Å] and the NCH_2_C CH group forms a dihedral angle of 67.95 (6)° with its best plane. In the crystal, mol­ecules form inversion dimers *via* pairs of N—H⋯O hydrogen bonds. C—H⋯O inter­actions connect the dimers, forming a two-dimensional polymeric network parallel to (100).

## Related literature

For pharmacological and biochemical properties of benzimidazole dirivatives, see: Gravatt *et al.* (1994 ▶); Horton *et al.* (2003 ▶); Kim *et al.* (1996 ▶); Roth *et al.* (1997 ▶). For similar structures, see: Ouzidan, Kandri Rodi, Butcher*et al.* (2011 ▶); Ouzidan, Kandri Rodi, Fronczek *et al.* (2011 ▶); Ouzidan, Kandri Rodi, Jasinski *et al.* (2011 ▶); Belaziz *et al.* (2012 ▶).
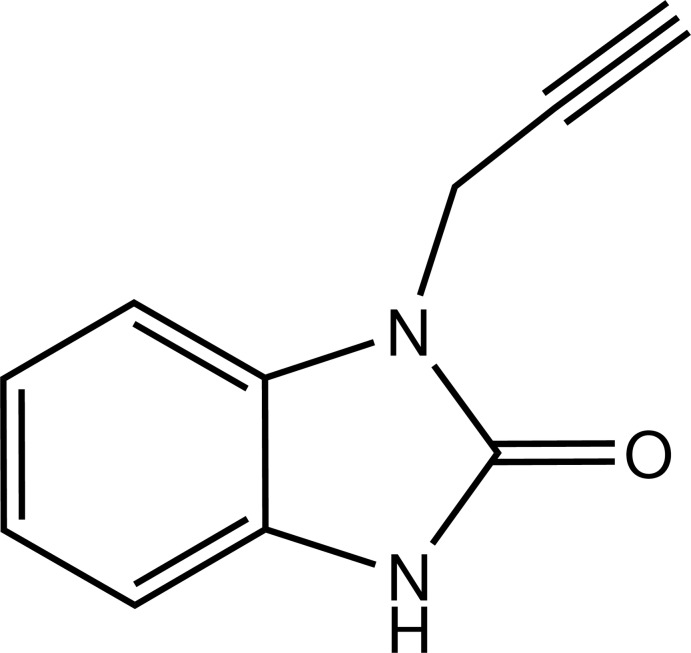


## Experimental

### 

#### Crystal data

C_10_H_8_N_2_O
*M* *_r_* = 172.18
Monoclinic, 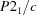

*a* = 4.5553 (6) Å
*b* = 18.001 (3) Å
*c* = 10.7488 (13) Å
β = 93.645 (8)°
*V* = 879.6 (2) Å^3^
*Z* = 4
Mo *K*α radiation
μ = 0.09 mm^−1^
*T* = 296 K
0.41 × 0.32 × 0.15 mm

#### Data collection

Bruker X8 APEXII diffractometer
10826 measured reflections
2080 independent reflections
1753 reflections with *I* > 2σ(*I*)
*R*_int_ = 0.020

#### Refinement

*R*[*F* ^2^ > 2σ(*F* ^2^)] = 0.035
*wR*(*F* ^2^) = 0.101
*S* = 1.05
2080 reflections
119 parameters
H-atom parameters constrained
Δρ_max_ = 0.16 e Å^−3^
Δρ_min_ = −0.16 e Å^−3^

### 

Data collection: *APEX2* (Bruker, 2009 ▶); cell refinement: *SAINT* (Bruker, 2009 ▶); data reduction: *SAINT*; program(s) used to solve structure: *SHELXS97* (Sheldrick, 2008 ▶); program(s) used to refine structure: *SHELXL97* (Sheldrick, 2008 ▶); molecular graphics: *ORTEP-3 for Windows* (Farrugia, 2012 ▶); software used to prepare material for publication: *PLATON* (Spek, 2009 ▶) and *publCIF* (Westrip, 2010 ▶).

## Supplementary Material

Crystal structure: contains datablock(s) I, global. DOI: 10.1107/S160053681205088X/gk2546sup1.cif

Structure factors: contains datablock(s) I. DOI: 10.1107/S160053681205088X/gk2546Isup2.hkl

Supplementary material file. DOI: 10.1107/S160053681205088X/gk2546Isup3.cml

Additional supplementary materials:  crystallographic information; 3D view; checkCIF report

## Figures and Tables

**Table 1:** Hydrogen-bond geometry (Å, °)

*D*—H⋯*A*	*D*—H	H⋯*A*	*D*⋯*A*	*D*—H⋯*A*
N1—H1⋯O1^i^	0.95	1.88	2.8226 (12)	174
C10—H10⋯O1^ii^	0.96	2.39	3.2541 (17)	149

Symmetry codes: (i) 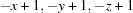; (ii) 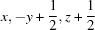.

**Fig. 1.**
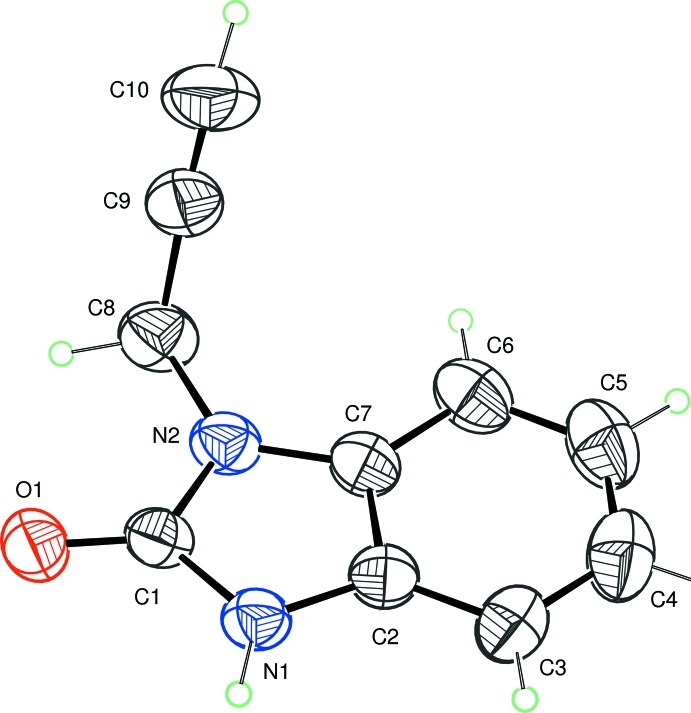
Molecular structure of the title compound with the atom-labelling scheme. Displacement ellipsoids are drawn at the 50% probability level. H atoms are represented as small circles.

**Fig. 2.**
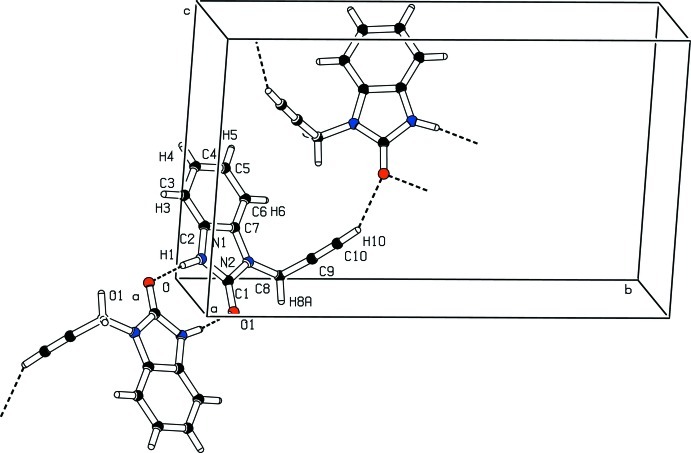
Intermolecular interactions in the title compound. Hydrogen bonds are shown as dashed lines. Symmetry codes: (i) -x + 1, -y + 1, -z + 1; (ii) x, -y + 1/2, z + 1/2.

## supplementary crystallographic information

### Comment

Benzimidazoles are very useful intermediates/subunits for the development of molecules of pharmaceutical or biological interest. Benzimidazole and its derivatives are an important class of bioactive molecules in the field of drugs and pharmaceuticals. Benzimidazole derivatives have found applications in diverse therapeutic areas including anti-ulcers, anti-hypertensives, anti-virals, anti-fungals, anti-cancers, (Gravatt *et al.*, 1994; Horton *et al.*, 2003; Kim *et al.*, 1996; Roth *et al.*, 1997).

As a continuation of our research work devoted to the development of substituted benzimidazol-2-one derivatives (Ouzidan, Kandri Rodi, Butcher*et al.*, 2011; Ouzidan, Kandri Rodi, Fronczek *et al.*, 2011; Belaziz *et al.* 2012), we reported in this paper the synthesis of benzimidazol-2-one derivative by action of propargyl bromide with 1*H*-benzo[*d*]imidazol-2(3*H*)-one in the presence of a catalytic quantity of tetra-n-butylammonium bromide under mild conditions to furnish two compounds: di-substituted (Ouzidan, Kandri Rodi, Jasinski *et al.*, 2011) and mono-substituted (Scheme 1).

The two fused five- and six-membered rings building the molecule of the title compound, C_10_H_8_N_2_O, are approximately planar, the largest deviation from the mean plane being 0.014 (2) Å at C1 (Fig.1). The C1–N2–C8–C9 torsion angle along the bond between the benzimidazolone and the prop-2-ynyl groups is -109.99 (12)°. In the crystal, the molecules form centrosymmetric cyclic dimers via a pair of N1–H1···O1 hydrogen-bonds (Fig.2 and Table2). In addition, intermolecular C10–H10···O1 interaction beteen the acetylenic H atom and the carbonyl O atom connects the dimers into (100) layers.

### Experimental

To 1*H*-benzo[*d*]imidazol-2(3*H*)-one (0.2 g, 1.5 mmol), potassium carbonate (0.41 g, 3 mmol) and tetra-n-butylammonium bromide (0.05 g, 0.15 mmol) in DMF (15 ml) was added propargyl bromide (0.16 ml, 1.8 mmol). Stirring was continued at room temperature for 6 h. The salt was removed by filtration and the filtrate concentrated under reduced pressure. The residue was separated by chromatography on a column of silica gel with ethyl acetate/petroleum ether (1/2) as eluent. Colourless crystals were isolated when the solvent was allowed to evaporate (m.p. 399 K).

### Refinement

All H atoms could be located in a difference Fourier map. However, they were placed in calculated positions with N—H = 0.86 Å, C—H = 0.93 Å (aromatic), and C—H = 0.97 Å (methylene) and refined as riding on their parent atoms with *U*_iso_(H) = 1.2 *U*_eq_ (C, N).

### Crystal data

**Table:**

C_10_H_8_N_2_O	*F*(000) = 360
*M**_r_* = 172.18	*D*_x_ = 1.300 Mg m^−^^3^
Monoclinic, *P*2_1_/*c*	Melting point: 399 K
Hall symbol: -P 2ybc	Mo *K*α radiation, λ = 0.71073 Å
*a* = 4.5553 (6) Å	Cell parameters from 2080 reflections
*b* = 18.001 (3) Å	θ = 3.0–27.9°
*c* = 10.7488 (13) Å	µ = 0.09 mm^−^^1^
β = 93.645 (8)°	*T* = 296 K
*V* = 879.6 (2) Å^3^	Block, colourless
*Z* = 4	0.41 × 0.32 × 0.15 mm

### Data collection

**Table:**

Bruker X8 APEXII diffractometer	1753 reflections with *I* > 2σ(*I*)
Radiation source: fine-focus sealed tube	*R*_int_ = 0.020
Graphite monochromator	θ_max_ = 27.9°, θ_min_ = 3.0°
φ and ω scans	*h* = −5→5
10826 measured reflections	*k* = −23→23
2080 independent reflections	*l* = −14→14

### Refinement

**Table:**

Refinement on *F*^2^	Secondary atom site location: difference Fourier map
Least-squares matrix: full	Hydrogen site location: difference Fourier map
*R*[*F*^2^ > 2σ(*F*^2^)] = 0.035	H-atom parameters constrained
*wR*(*F*^2^) = 0.101	*w* = 1/[σ^2^(*F*_o_^2^) + (0.0493*P*)^2^ + 0.1197*P*] where *P* = (*F*_o_^2^ + 2*F*_c_^2^)/3
*S* = 1.05	(Δ/σ)_max_ < 0.001
2080 reflections	Δρ_max_ = 0.16 e Å^−^^3^
119 parameters	Δρ_min_ = −0.16 e Å^−^^3^
0 restraints	Extinction correction: *SHELXL97* (Sheldrick, 2008), Fc^*^=kFc[1+0.001xFc^2^λ^3^/sin(2θ)]^-1/4^
Primary atom site location: structure-invariant direct methods	Extinction coefficient: 0.018 (4)

### Special details

**Table:**

Geometry. All e.s.d.'s (except the e.s.d. in the dihedral angle between two l.s. planes) are estimated using the full covariance matrix. The cell e.s.d.'s are taken into account individually in the estimation of e.s.d.'s in distances, angles and torsion angles; correlations between e.s.d.'s in cell parameters are only used when they are defined by crystal symmetry. An approximate (isotropic) treatment of cell e.s.d.'s is used for estimating e.s.d.'s involving l.s. planes.
Refinement. Refinement of *F*^2^ against all reflections. The weighted *R*-factor *wR* and goodness of fit *S* are based on *F*^2^, conventional *R*-factors *R* are based on *F*, with *F* set to zero for negative *F*^2^. The threshold expression of *F*^2^ > σ(*F*^2^) is used only for calculating *R*-factors(gt) *etc*. and is not relevant to the choice of reflections for refinement. *R*-factors based on *F*^2^ are statistically about twice as large as those based on *F*, and *R*- factors based on all data will be even larger.

### Fractional atomic coordinates and isotropic or equivalent isotropic displacement parameters (Å^2^)

**Table:**

	*x*	*y*	*z*	*U*_iso_*/*U*_eq_	
O1	0.43254 (19)	0.40104 (4)	0.43888 (7)	0.0527 (2)	
N1	0.25564 (19)	0.46700 (5)	0.60486 (8)	0.0425 (2)	
H1	0.3557	0.5125	0.5955	0.051*	
N2	0.0986 (2)	0.35310 (5)	0.57304 (8)	0.0436 (2)	
C1	0.2795 (2)	0.40708 (5)	0.52935 (9)	0.0412 (2)	
C2	0.0662 (2)	0.45135 (6)	0.69742 (9)	0.0412 (2)	
C3	−0.0238 (3)	0.49315 (7)	0.79538 (11)	0.0523 (3)	
H3	0.0420	0.5416	0.8087	0.063*	
C4	−0.2163 (3)	0.46000 (9)	0.87330 (12)	0.0623 (4)	
H4	−0.2806	0.4868	0.9403	0.075*	
C5	−0.3150 (3)	0.38814 (9)	0.85397 (12)	0.0625 (4)	
H5	−0.4446	0.3677	0.9080	0.075*	
C6	−0.2248 (3)	0.34587 (7)	0.75573 (11)	0.0543 (3)	
H6	−0.2904	0.2974	0.7427	0.065*	
C7	−0.0329 (2)	0.37902 (6)	0.67788 (9)	0.0421 (3)	
C8	0.0605 (3)	0.27990 (6)	0.51808 (11)	0.0539 (3)	
H8A	0.1497	0.2789	0.4385	0.065*	
H8B	−0.1478	0.2697	0.5029	0.065*	
C9	0.1941 (3)	0.22211 (6)	0.59936 (12)	0.0553 (3)	
C10	0.2989 (4)	0.17785 (7)	0.66881 (15)	0.0737 (4)	
H10	0.3795	0.1424	0.7281	0.088*	

### Atomic displacement parameters (Å^2^)

**Table:**

	*U*^11^	*U*^22^	*U*^33^	*U*^12^	*U*^13^	*U*^23^
O1	0.0709 (5)	0.0453 (4)	0.0429 (4)	−0.0091 (4)	0.0121 (4)	−0.0023 (3)
N1	0.0500 (5)	0.0341 (4)	0.0435 (5)	−0.0036 (3)	0.0030 (4)	0.0009 (3)
N2	0.0516 (5)	0.0355 (4)	0.0434 (5)	−0.0065 (4)	0.0002 (4)	0.0010 (3)
C1	0.0490 (6)	0.0359 (5)	0.0381 (5)	−0.0023 (4)	−0.0020 (4)	0.0038 (4)
C2	0.0389 (5)	0.0424 (5)	0.0415 (5)	0.0029 (4)	−0.0029 (4)	0.0030 (4)
C3	0.0491 (6)	0.0549 (7)	0.0525 (6)	0.0059 (5)	0.0004 (5)	−0.0075 (5)
C4	0.0502 (7)	0.0862 (10)	0.0506 (7)	0.0099 (6)	0.0061 (5)	−0.0070 (6)
C5	0.0464 (6)	0.0881 (10)	0.0536 (7)	−0.0025 (6)	0.0081 (5)	0.0120 (6)
C6	0.0478 (6)	0.0594 (7)	0.0555 (7)	−0.0086 (5)	0.0009 (5)	0.0114 (5)
C7	0.0401 (5)	0.0444 (6)	0.0411 (5)	−0.0010 (4)	−0.0038 (4)	0.0049 (4)
C8	0.0690 (8)	0.0412 (6)	0.0505 (6)	−0.0135 (5)	−0.0032 (5)	−0.0032 (5)
C9	0.0709 (8)	0.0365 (6)	0.0590 (7)	−0.0124 (5)	0.0083 (6)	−0.0038 (5)
C10	0.0977 (11)	0.0425 (7)	0.0799 (10)	−0.0040 (7)	−0.0020 (8)	0.0087 (6)

### Geometric parameters (Å, º)

**Table:**

N1—C1	1.3583 (13)	C8—H8A	0.9700
N1—C2	1.3869 (13)	C8—H8B	0.9700
N1—H1	0.9458	C9—C10	1.1725 (19)
N2—C1	1.3760 (13)	C3—C4	1.3856 (18)
N2—C7	1.3900 (14)	C3—H3	0.9300
N2—C8	1.4500 (13)	C6—C5	1.3848 (19)
C2—C3	1.3775 (15)	C6—H6	0.9300
C2—C7	1.3894 (15)	C5—C4	1.381 (2)
O1—C1	1.2367 (13)	C5—H5	0.9300
C7—C6	1.3835 (15)	C4—H4	0.9300
C8—C9	1.4657 (17)	C10—H10	0.9580
			
C1—N1—C2	110.15 (9)	N2—C8—H8B	109.3
C1—N1—H1	124.5	C9—C8—H8B	109.3
C2—N1—H1	125.3	H8A—C8—H8B	108.0
C1—N2—C7	109.75 (9)	C10—C9—C8	177.04 (14)
C1—N2—C8	124.15 (9)	C2—C3—C4	117.20 (12)
C7—N2—C8	126.08 (9)	C2—C3—H3	121.4
C3—C2—N1	131.78 (10)	C4—C3—H3	121.4
C3—C2—C7	121.20 (10)	C7—C6—C5	116.98 (12)
N1—C2—C7	107.02 (9)	C7—C6—H6	121.5
C6—C7—C2	121.62 (10)	C5—C6—H6	121.5
C6—C7—N2	131.84 (10)	C4—C5—C6	121.32 (12)
C2—C7—N2	106.54 (9)	C4—C5—H5	119.3
O1—C1—N1	127.61 (9)	C6—C5—H5	119.3
O1—C1—N2	125.87 (9)	C5—C4—C3	121.67 (12)
N1—C1—N2	106.52 (9)	C5—C4—H4	119.2
N2—C8—C9	111.58 (9)	C3—C4—H4	119.2
N2—C8—H8A	109.3	C9—C10—H10	177.7
C9—C8—H8A	109.3		
			
C1—N1—C2—C3	178.95 (11)	C8—N2—C1—O1	−0.04 (17)
C1—N1—C2—C7	−0.44 (11)	C7—N2—C1—N1	−1.39 (11)
C3—C2—C7—C6	−0.13 (16)	C8—N2—C1—N1	−179.94 (9)
N1—C2—C7—C6	179.33 (9)	C1—N2—C8—C9	109.99 (12)
C3—C2—C7—N2	−179.88 (9)	C7—N2—C8—C9	−68.32 (15)
N1—C2—C7—N2	−0.42 (11)	N1—C2—C3—C4	−179.25 (10)
C1—N2—C7—C6	−178.59 (11)	C7—C2—C3—C4	0.07 (16)
C8—N2—C7—C6	−0.07 (18)	C2—C7—C6—C5	0.22 (16)
C1—N2—C7—C2	1.13 (11)	N2—C7—C6—C5	179.90 (11)
C8—N2—C7—C2	179.65 (10)	C7—C6—C5—C4	−0.25 (18)
C2—N1—C1—O1	−178.79 (10)	C6—C5—C4—C3	0.2 (2)
C2—N1—C1—N2	1.12 (11)	C2—C3—C4—C5	−0.10 (18)
C7—N2—C1—O1	178.52 (10)		

### Hydrogen-bond geometry (Å, º)

**Table:**

*D*—H···*A*	*D*—H	H···*A*	*D*···*A*	*D*—H···*A*
N1—H1···O1^i^	0.95	1.88	2.8226 (12)	174
C10—H10···O1^ii^	0.96	2.39	3.2541 (17)	149

Symmetry codes: (i) −*x*+1, −*y*+1, −*z*+1; (ii) *x*, −*y*+1/2, *z*+1/2.

## References

[bb1] Belaziz, D., Kandri Rodi, Y., Ouazzani Chahdi, F., Essassi, E. M., Saadi, M. & El Ammari, L. (2012). *Acta Cryst.* E**68**, o3212. 10.1107/S1600536812043620 PMC3515299 23284519

[bb2] Bruker (2009). *APEX2* and *SAINT* Bruker AXS Inc., Madison, Wisconsin, USA.

[bb3] Farrugia, L. J. (2012). *J. Appl. Cryst.* **45**, 849–854.

[bb4] Gravatt, G. L., Baguley, B. C., Wilson, W. R. & Denny, W. A. (1994). *J. Med. Chem.* **37**, 4338–4345. 10.1021/jm00051a010 7527862

[bb5] Horton, D. A., Bourne, G. T. & Smythe, M. L. (2003). *Chem. Rev.* **103**, 893–930. 10.1021/cr020033s 12630855

[bb6] Kim, J. S., Gatto, B., Yu, C., Liu, A., Liu, L. F. & La Voie, E. J. (1996). *J. Med. Chem.* **39**, 992–998. 10.1021/jm950412w 8632422

[bb7] Ouzidan, Y., Kandri Rodi, Y., Butcher, R. J., Essassi, E. M. & El Ammari, L. (2011). *Acta Cryst.* E**67**, o283. 10.1107/S1600536810054164 PMC3051596 21522975

[bb8] Ouzidan, Y., Kandri Rodi, Y., Fronczek, F. R., Venkatraman, R., El Ammari, L. & Essassi, E. M. (2011). *Acta Cryst.* E**67**, o362–o363. 10.1107/S1600536810052141 PMC3051530 21523041

[bb9] Ouzidan, Y., Kandri Rodi, Y., Jasinski, J. P., Butcher, R. J., Golen, J. A. & El Ammari, L. (2011). *Acta Cryst.* E**67**, o1091. 10.1107/S1600536811012578 PMC3089259 21754411

[bb10] Roth, T., Morningstar, M. L., Boyer, P. L., Hughes, S. H., Buckheit, R. W. & Michejda, C. J. (1997). *J. Med. Chem.* **40**, 4199–4207. 10.1021/jm970096g 9435891

[bb11] Sheldrick, G. M. (2008). *Acta Cryst.* A**64**, 112–122. 10.1107/S0108767307043930 18156677

[bb12] Spek, A. L. (2009). *Acta Cryst.* D**65**, 148–155. 10.1107/S090744490804362X PMC2631630 19171970

[bb13] Westrip, S. P. (2010). *J. Appl. Cryst.* **43**, 920–925.

